# Prognostic value of admission ionized calcium for short-term mortality in critically Ill children with sepsis: a single-center retrospective cohort study

**DOI:** 10.3389/fped.2026.1793547

**Published:** 2026-05-15

**Authors:** Man Chen, Juan Feng, Zongwei Zhang, Xifeng Lv, Ming Shi

**Affiliations:** 1 Division of Nephrology, Renmin Hospital of Wuhan University, Wuhan, China; 2 Department of Medical Intensive Care Unit, Maternal and Child Health Hospital of Hubei Province, Tongji Medical College, Huazhong University of Science and Technology, Wuhan, China

**Keywords:** 28-day mortality, hypocalcemia, ionized calcium, pediatric intensive care unit, pediatric sepsis, prognostic value

## Abstract

**Background:**

Sepsis remains a leading cause of morbidity and mortality among critically ill children admitted to pediatric intensive care units (PICUs). Early risk stratification at admission is essential but is often limited by the complexity and delayed availability of existing severity scoring systems. Disturbances in calcium homeostasis, particularly hypocalcemia, are common in critical illness; however, the prognostic significance of admission serum ionized calcium (iCa) for short-term mortality in pediatric sepsis has not been well defined.

**Methods:**

This single-center retrospective cohort study analyzed data from the Pediatric Intensive Care database of the Children's Hospital affiliated with Zhejiang University School of Medicine (2010–2019). Children with sepsis who had iCa measurements within 24 h of PICU admission were included. Hypocalcemia was defined as iCa < 1.15 mmol/L. The primary outcome was 28-day all-cause mortality. Kaplan–Meier analysis and Cox proportional hazards models were used to assess the association between iCa and mortality. Discriminatory performance and incremental prognostic value were evaluated using receiver operating characteristic (ROC) curves, integrated discrimination improvement (IDI), and net reclassification improvement (NRI). Subgroup analyses and interaction tests were performed to assess the consistency of the association between iCa and mortality across predefined strata of disease severity.

**Results:**

A total of 289 children were included, among whom 26 (9.0%) died within 28 days. Hypocalcemia at admission was common (50.8%) and was associated with significantly higher mortality compared with normocalcemia (19.2% vs. 3.4%; log-rank *p* < 0.001). In univariable analysis, each 0.1 mmol/L decrease in iCa was associated with increased mortality risk (HR 1.53, 95% CI 1.30–1.80). This association remained significant after adjustment for vasoactive agent use and lactate (adjusted HR 1.55, 95% CI 1.29–1.86). Admission iCa demonstrated moderate discrimination for 28-day mortality (AUC 0.734), with high sensitivity (88.5%) at the predefined clinical threshold. Adding iCa to the baseline clinical model improved risk reclassification (IDI 0.116; NRI 0.324), although the increase in AUC was not statistically significant. Subgroup analyses showed the association was more pronounced in patients with lactate ≤ 4 mmol/L and those not receiving vasoactive support (interaction *p* < 0.05).

**Conclusions:**

Lower admission iCa levels are independently associated with higher 28-day mortality in pediatric sepsis, particularly in the early phase of illness. As a rapid and routinely available biomarker, iCa may enhance early risk stratification in critically ill children with sepsis.

## 1 Background

Sepsis remains one of the most common and life-threatening conditions encountered in pediatric intensive care units (PICUs), particularly in cases of septic shock, which are characterized by rapid clinical deterioration and high mortality. Globally, approximately 1.2 million cases of pediatric sepsis occur each year, with reported mortality rates ranging from 6% to 25%, while mortality associated with septic shock may reach up to 50% (1–4). Despite substantial advances in critical care and antimicrobial therapy, outcomes in children with sepsis remain suboptimal. Early identification of high-risk patients and timely intervention are therefore essential for improving prognosis. Currently, disease severity in pediatric sepsis is commonly assessed using scoring systems such as the pediatric Sequential Organ Failure Assessment (pSOFA) score and the Pediatric Risk of Mortality (PRISM III) score. However, these tools often rely on dynamic monitoring after admission and require multiple variables, which may limit their practicality for rapid risk stratification at initial presentation (5–7). Therefore, identifying simple, objective, and readily available biomarkers that could complement existing scoring systems or provide early prognostic information may be of clinical value. Electrolyte disturbances are frequent pathophysiological abnormalities in critically ill children, among which disruption of calcium homeostasis is particularly prominent (8). Serum ionized calcium (iCa) represents the biologically active fraction of circulating calcium and plays a pivotal role in essential physiological processes, including coagulation, neuromuscular excitability, myocardial contraction, and intracellular signal transduction (9). In the setting of critical illness, hypocalcemia is driven by a complex interplay of factors. A key mechanism is a transcellular shift, wherein massive catecholamine release, metabolic stress, and systemic inflammation during sepsis promote calcium influx into cells (10). This process is often compounded by suppressed parathyroid hormone activity and impaired vitamin D metabolism (11). As early as 1987, Broner et al. reported that hypocalcemia was present in up to 12.5% of critically ill children at admission and was associated with a significantly higher mortality rate (46%) compared with normocalcemic (9.5%) and hypercalcemic (0%) patients (*p* < 0.001) (12). Subsequent studies have further demonstrated a close association between iCa levels and disease severity in critically ill children (13–15). In very low birth weight infants with sepsis, lower iCa levels were independently associated with increased neonatal mortality (32.8% vs. 12.8%, *p* < 0.001) and identified as an independent predictor of adverse outcomes (OR = 0.558, 95% CI 0.406–0.768, *p* < 0.001) (14). Additional investigations have shown that both total calcium and iCa levels decrease during the acute phase of critical illness, with a more pronounced reduction in iCa, and that lower iCa levels are inversely correlated with illness severity scores (8, 11, 13, 16).

Nevertheless, most available evidence has focused on neonates or very low birth weight infants with sepsis (12, 14), or on heterogeneous populations of critically ill children where sepsis is only a subgroup (16). While these studies are valuable, they either limit generalizability to older children or do not allow for conclusions specific to the pathophysiology and trajectory of pediatric sepsis. Data specifically addressing the relationship between admission iCa levels and short-term mortality, such as 28-day mortality, in a well-defined cohort of children with sepsis remain limited. Because admission laboratory parameters are readily obtainable and reflect early pathophysiological derangements, biomarkers capable of providing prognostic information at this stage may offer substantial clinical value. Therefore, this single-center retrospective study aimed to investigate the association between admission serum iCa levels and 28-day all-cause mortality in children with sepsis, and to evaluate its potential as an early prognostic biomarker for risk stratification in this high-risk population.

## 2 Methods

### 2.1 Study design and data source

This study was conducted as a single-center retrospective cohort study using data derived from the Pediatric Intensive Care database (PIC database) established at the Children's Hospital affiliated with Zhejiang University School of Medicine (17). The PIC database was originally created to systematically collect comprehensive clinical data from critically ill children, with the primary aim of supporting clinical research on disease patterns, treatment effectiveness, and outcome prediction in pediatric intensive care. The database contains comprehensive clinical information on critically ill children admitted to the PICU between 2010 and 2019, including 13,941 hospitalization records from 12,881 unique patients. The database integrates structured data on demographics, vital signs, laboratory test results, therapeutic interventions, and clinical outcomes. The present study was performed after signing a restricted data use agreement and obtaining formal authorization. All data were fully de-identified prior to inclusion in the database, with no personally identifiable information available. In accordance with institutional and ethical regulations, the requirement for informed consent from patients or their legal guardians was waived.

### 2.2 Study population

**Figure 1:**
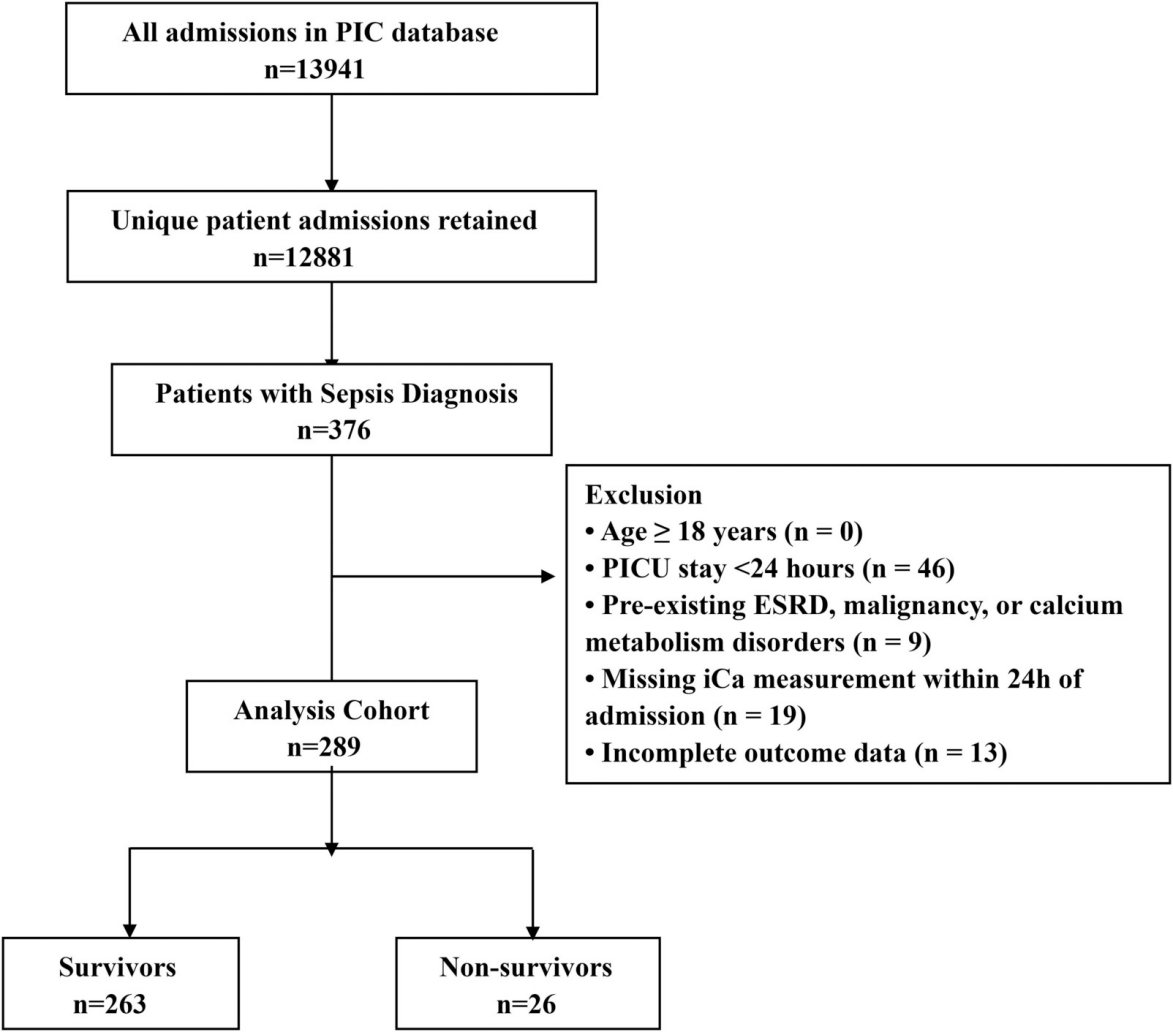
Cohort selection flowchart. PICU, Pediatric Intensive Care Unit; ICD-10, International Classification of Diseases, Tenth Revision; ESRD, End-Stage Renal Disease; iCa, Ionized Calcium.

The study population was derived from all PICU admissions recorded in the PIC database. A total of 13,941 PICU admission records were initially extracted, and after excluding duplicate hospitalizations, 12,881 unique patients were identified. Children meeting the diagnostic criteria for sepsis were identified using the International Classification of Diseases, Tenth Revision (ICD-10) codes. Importantly, no restriction was placed on the position of the diagnosis; patients were included regardless of whether sepsis was recorded as the primary admission diagnosis or as a secondary diagnosis during hospitalization. Thus, this cohort reflects the real-world clinical scenario in the PICU, encompassing both children admitted primarily for sepsis and those who developed sepsis secondary to underlying diseases. Given that underlying comorbidities may influence both calcium homeostasis and clinical outcomes, we applied the following exclusion criteria to minimize potential confounding bias: age ≥ 18 years; PICU length of stay < 24 h; pre-existing end-stage renal disease, malignant tumors, or known conditions affecting calcium metabolism (e.g., primary parathyroid disorders or severe vitamin D metabolism disorders); missing serum ionized calcium (iCa) measurements within the first 24 h of PICU admission; or incomplete outcome data. The detailed patient selection process is illustrated in Figure 1.

### 2.3 Data collection and variable definitions

Study data were extracted from relevant PIC database tables using structured query language (SQL). Demographic characteristics (age and sex) were obtained from the Patients table. Admission information and clinical outcomes were retrieved from the Admissions and ICU stays tables. Laboratory test results were obtained from the LabEvents table. All laboratory variables were defined as the first measured values within 24 h of PICU admission. Collected variables included demographic characteristics; indicators of disease severity (use of vasoactive agents and initial arterial blood lactate level); and laboratory parameters, including white blood cell count (WBC), neutrophil count, platelet count (PLT), serum ionized calcium(iCa), sodium (Na⁺), potassium (K⁺), chloride (Cl⁻), serum creatinine, blood urea nitrogen (BUN), albumin, alanine aminotransferase (ALT), aspartate aminotransferase (AST), total bilirubin, and C-reactive protein (CRP). Due to the structure of the database, detailed information on underlying comorbidities and infection sources was not consistently available; therefore, these variables were not included in the analysis. iCa levels were expressed in mmol/L. In routine clinical practice at the study institution, iCa was measured using arterial or venous blood samples analyzed by automated blood gas analyzers based on ion-selective electrode methodology, following standardized laboratory quality control procedures. Based on commonly accepted clinical reference thresholds, hypocalcemia was defined as an iCa level < 1.15 mmol/L (10).

### 2.4 Outcome measures

The primary outcome of this study was 28-day all-cause mortality, defined as death from any cause occurring within 28 days after PICU admission, regardless of whether the patient remained hospitalized. Survival time was calculated from the date of PICU admission to the date of death or the end of the 28-day follow-up period, whichever occurred first.

### 2.5 Statistical analysis

All statistical analyses were performed using Decision Chain software. Continuous variables were first assessed for normality; those with a normal distribution are presented as mean ± standard deviation and were compared between groups using the independent-samples t test, whereas non-normally distributed variables are expressed as median (interquartile range) and were compared using the Mann–Whitney U test. Categorical variables are presented as counts (percentages) and were compared using the chi-square test or Fisher's exact test, as appropriate. Kaplan–Meier curves were constructed to estimate survival probability over the 28-day follow-up period stratified by the predefined clinical threshold for hypocalcemia (iCa < 1.15 mmol/L), and differences between groups were assessed using the log-rank test. Cox proportional hazards regression models were applied to evaluate the association between serum iCa levels and 28-day mortality, with results reported as hazard ratios (HRs) and corresponding 95% confidence intervals (CIs). The proportional hazards assumption was tested by examining Schoenfeld residuals. Multicollinearity among covariates was assessed using the variance inflation factor (VIF), with lower VIF values indicating less collinearity; a VIF < 5 is generally considered acceptable. Given the limited number of outcome events (*n* = 26), we adopted a parsimonious modeling strategy to minimize the risk of overfitting. Based on the rule of thumb recommending at least 10 events per predictor variable, the multivariable Cox model included serum iCa and two key indicators of disease severity: use of vasoactive agents (reflecting cardiovascular dysfunction) and initial lactate level (reflecting tissue hypoperfusion). These variables were selected due to their established prognostic value in pediatric sepsis and their central role in the pathophysiological cascade of septic shock. To assess the robustness of our findings, we conducted two sensitivity analyses. First, we replaced lactate with blood urea nitrogen (BUN) in the multivariable Cox model while retaining vasoactive agent use. Second, we constructed an expanded model that simultaneously included iCa, vasoactive agent use, lactate, and BUN. These analyses allowed us to determine whether the association between iCa and 28-day mortality remained consistent when adjusting for different markers of organ dysfunction. To evaluate the discriminatory performance of serum iCa for predicting 28-day mortality, we performed receiver operating characteristic (ROC) curve analysis and calculated the area under the curve (AUC). The sensitivity and specificity of the clinically predefined hypocalcemia threshold (iCa < 1.15 mmol/L) were also reported. To assess the incremental prognostic value of iCa, a baseline clinical prediction model was constructed using vasoactive agent use and lactate; the area under the receiver operating characteristic curve (AUC) was compared before and after the inclusion of iCa. Differences in AUCs were evaluated using the DeLong test, and improvements in model performance were further quantified using the integrated discrimination improvement (IDI) and net reclassification improvement (NRI) indices. Subgroup analyses were performed using univariable Cox regression models within each stratum, including only serum iCa as the predictor, to evaluate its association with 28-day mortality. To formally assess effect modification, interaction terms between iCa and predefined subgroup variables (iCa × subgroup variable) were introduced into Cox proportional hazards models. These subgroups were selected based on clinical relevance: age reflects developmental differences in calcium homeostasis; lactate level indicates the severity of tissue hypoperfusion; and vasoactive agent use serves as a marker of cardiovascular dysfunction. Evaluating the consistency of the iCa–mortality association across these subgroups may help identify populations in which iCa provides stronger or weaker prognostic informationAll statistical tests were two-sided, and a P value < 0.05 was considered statistically significant.

## 3 Results

### 3.1 Baseline characteristics of the study cohort

A total of 289 children meeting the diagnostic criteria for sepsis and requiring PICU admission were included in the analysis (Table 1). The median age of the cohort was 0.19 years (IQR: 0.03–1.81), and 173 patients were male (59.9%). During the 28-day follow-up period, 26 deaths occurred, corresponding to an overall mortality rate of 9.0%. As summarized in Table 1, significant differences in disease severity and organ function at PICU admission were observed between survivors and non-survivors. Compared with survivors, non-survivors were more likely to require vasoactive agent support (56.0% vs. 25.0%, *p* < 0.001) and had higher initial blood lactate levels (2.90 vs. 1.95 mmol/L, *p* = 0.003). Among the entire cohort, 69 patients (23.9%) presented with an initial lactate level > 4 mmol/L; in this subgroup, 11 deaths occurred, yielding a mortality rate of 15.9% (Supplementary Table S1). Laboratory assessments showed that non-survivors had generally higher levels of markers of organ dysfunction and inflammatory markers, including serum creatinine (77.35 vs. 46.00 μmol/L, *p* = 0.016), blood urea nitrogen (7.39 vs. 3.69 mmol/L, *p* < 0.001), alanine aminotransferase (62.50 vs. 19.00 U/L, *p* < 0.001), aspartate aminotransferase (153.00 vs. 43.50 U/L, *p* < 0.001), and C-reactive protein (53.50 vs. 31.00 mg/L, *p* = 0.037), whereas serum albumin levels were significantly lower (26.98 vs. 31.29 g/L, *p* = 0.012). Notably, admission serum iCa levels were significantly lower in non-survivors than in survivors (median: 1.02 mmol/L [IQR: 0.90–1.12] vs. 1.16 mmol/L [IQR: 1.05–1.24], *p* < 0.001).

**Table 1:** Baseline characteristics of pediatric patients stratified by 28-Day mortality Status (*N* = 289).

Characteristic	Total (*n* = 289)	Survivors (*n* = 263)	Non-survivors (*n* = 26)	*p*-value
Demographics				
Age (years)	0.19 (0.03,1.81)	0.19 (0.03,1.81)	0.31 (0.10,2.21)	0.392
Male, *n* (%)	173 (59.86%)	156 (59.32%)	17 (65.38%)	0.547
Clinical severity				
Vasopressor use, n (%)	77 (27.80%)	63 (25.00%)	14 (56.00%)	<0.001
Lactate (mmol/L)	2.00 (1.30,3.90)	1.95 (1.30,3.75)	2.90 (2.10,6.60)	0.003
Laboratory parameters				
WBC (10⁹/L)	9.90 (5.31,15.29)	10.01 (6.02,14.94)	7.88 (2.45,16.09)	0.441
Neutrophil (10⁹/L)	5.30 (2.36,10.23)	5.45 (2.56,10.23)	3.80 (0.85,10.27)	0.126
PLT (10⁹/L)	191.00 (91.00,305.00)	193.00 (95.00,308.00)	142.00 (57.00,231.00)	0.122
Creatinine (umol/L)	48.00 (37.00,75.00)	46.00 (36.00,70.00)	77.35 (40.40,108.00)	0.016
BUN (mmol/L)	3.99 (2.62,6.80)	3.69 (2.54,6.06)	7.39 (5.47,10.50)	<0.001
Albumin (g/L)	30.90 (6.87)	31.29 (6.65)	26.98 (7.94)	0.012
ALT (U/L)	20.00 (11.00,53.00)	19.00 (11.00,44.00)	62.50 (18.00,162.00)	<0.001
AST (U/L)	47.50 (27.00,111.00)	43.50 (26.00,96.00)	153.00 (65.00,421.00)	<0.001
Total Bilirubin (umol/L)	22.70 (8.20,94.40)	23.30 (8.20,92.70)	21.00 (6.50,94.40)	0.984
CRP (mg/L),	32.19 (10.00,85.60)	31.00 (9.00,80.02)	53.50 (25.00,111.00)	0.037
K (mmol/L)	3.80 (3.30,4.30)	3.80 (3.30,4.30)	4.00 (2.70,4.60)	0.947
Na (mmol/L)	136.00 (132.00,139.00)	136.00 (132.00,139.00)	136.00 (129.00,140.00)	0.933
Cl (mmol/L)	109.00 (105.00,112.00)	109.00 (105.00,112.00)	107.50 (104.00,111.00)	0.504
iCa (mmol/L)	1.14 (1.03,1.03)	1.16 (1.05,1.24)	1.02 (0.90,1.12)	<0.001

Values are presented as mean ± SD for normally distributed continuous variables, median (IQR) for non-normally distributed continuous variables, and *n* (%) for categorical variables. WBC, white blood cell; PLT, platelet count; BUN, blood urea; ALT, Alanine aminotransferase; AST, aspartate aminotransferase; CRP, C-Reactive Protein; iCa, ionized calcium.

### 3.2 Association between serum iCa levels and 28-day mortality

**Figure 2:**
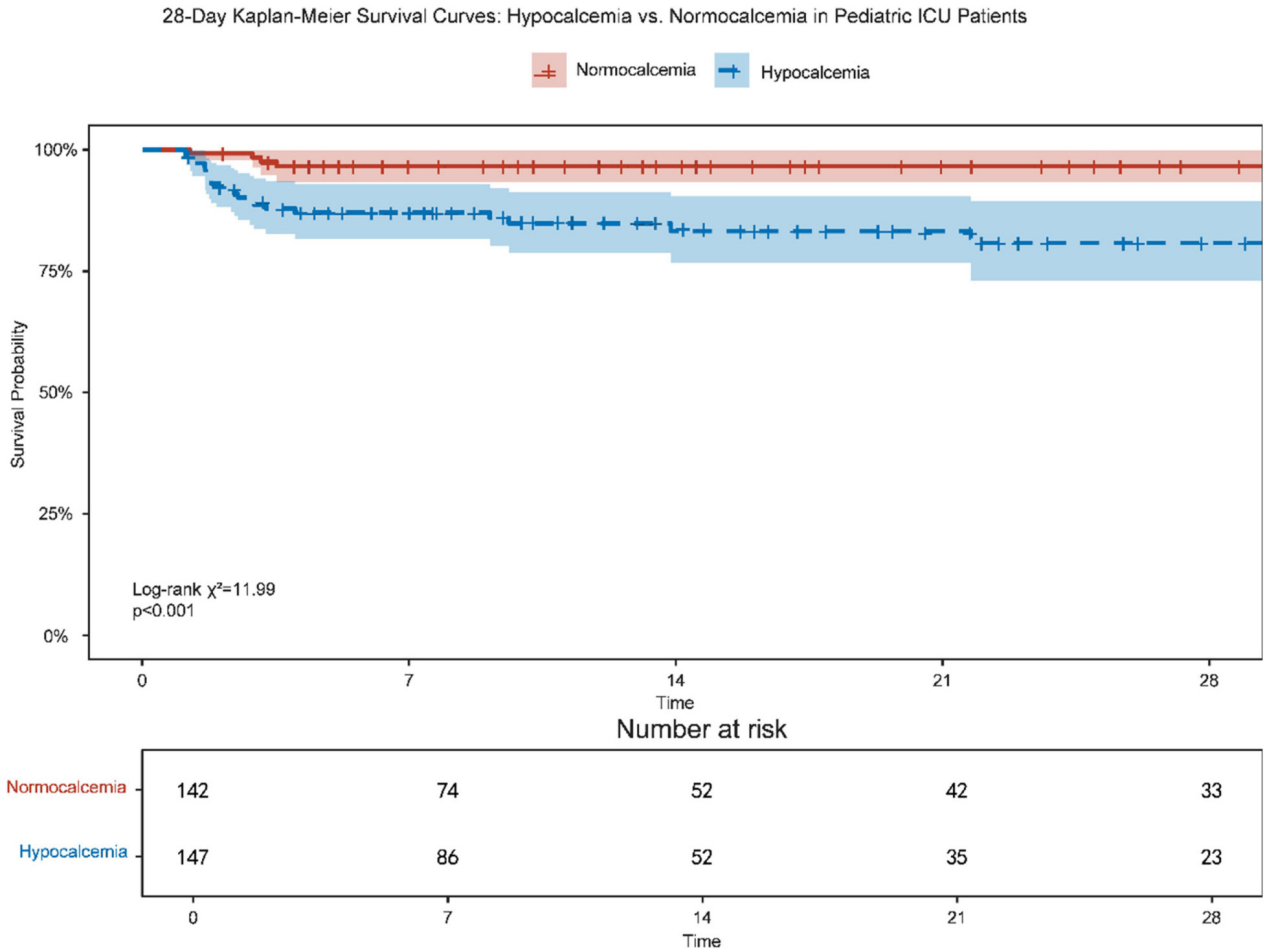
Kaplan–Meier survival curves for 28-day survival probability in pediatric ICU patients with hypocalcemia versus normocalcemia.

Based on a clinically relevant threshold (serum iCa < 1.15 mmol/L), patients were classified into a hypocalcemia group (*n* = 147) and a non-hypocalcemia group (*n* = 142). Kaplan–Meier survival analysis demonstrated a significant difference in 28-day survival probability between the hypocalcemia and non-hypocalcemia groups (Figure 2). The 28-day mortality rate was significantly higher in the hypocalcemia group than in the non-hypocalcemia group (19.2% vs. 3.4%; log-rank *p* < 0.001). In univariable Cox proportional hazards analysis, each 0.1 mmol/L decrease in serum iCa was associated with a 53% increase in the risk of death (HR = 1.53, 95% CI: 1.30–1.80, *p* < 0.001; Table 2).

**Table 2:** Cox regression analysis of serum ionized calcium and 28-day mortality risk.

Variable	HR/aHR (95% CI)	*P*-value
A. Univariate analysis		
iCa (per 0.1 mmol/L decrease)	1.53 (1.30–1.80)	<0.001
Vasopressor use	3.39 (1.54–7.48)	0.002
Lactate (per 1 mmol/L increase)	1.11 (1.02–1.21)	0.015
Creatinine (per 1 μmol/L increase)	1.01 (1.00–1.02)	0.010
Albumin (per 1 g/L decrease)	0.93 (0.88–0.98)	0.006
BUN (per 1 mmol/L increase)	1.15 (1.07–1.23)	<0.001
ALT (per 1 U/L increase)	1.00 (1.00–1.00)	0.057
AST (per 1 U/L increase)	1.00 (1.00–1.00)	0.001
CRP (per 1 mg/L increase)	1.01 (0.99–1.01)	0.126
B. Multivariable analysis		
iCa (per 0.1 mmol/L decrease)	1.55 (1.29–1.86)	<0.001

HR, hazard ratio; aHR, adjusted hazard ratio.

**Table 3:** Forest plot of subgroup and interaction analysis: iCa & mortality risk.

Variable	HR (95%CI)	*P*_value		*P*_for_interaction
Overall	1.51 (1.27–1.79)	<0.001	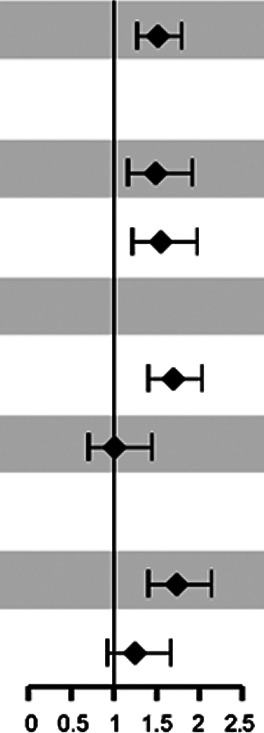	
Age				0.935
<1 year	1.48 (1.16–1.91)	0.002		
≥1 year	1.54 (1.21, 1.97)	<0.001		
Lactate (mmol/L)				0.014
≤4	1.69 (1.40–2.03)	<0.001		
>4	1.00 (0.70–1.44)	0.989		
Vasoactive_drug				0.061
Not used	1.73 (1.40–2.14)	<0.001		
Used	1.24 (0.92–1.66)	0.156		

### 3.3 Independent prognostic value of serum ICa for 28-day mortality

In univariable analyses, multiple clinical and laboratory parameters, including vasoactive agent use, initial lactate level, serum creatinine, blood urea nitrogen, aspartate aminotransferase, and serum albumin, were significantly associated with 28-day mortality (all *p* < 0.05; Table 2). To minimize the risk of model overfitting given the limited number of outcome events (*n* = 26), we adopted a parsimonious modeling strategy. The multivariable Cox proportional hazards model included serum iCa along with two key indicators of disease severity—vasoactive agent use and initial lactate level—selected based on their established prognostic value and central role in the pathophysiological cascade of septic shock. After adjustment, serum iCa remained an independent predictor of 28-day all-cause mortality, with each 0.1 mmol/L decrease associated with a significantly increased risk of death (adjusted HR = 1.55, 95% CI: 1.29–1.86, *p* < 0.001; Table 2). Schoenfeld residual tests showed no significant violations of the proportional hazards assumption for any variables included in the Cox regression model (all *p* > 0.05) (Supplementary Table S2). Collinearity diagnostics demonstrated low multicollinearity among covariates, with all variance inflation factors (VIFs) below 2, including lactate and vasoactive agent use, indicating good model stability (Supplementary Table S3).

The sensitivity analyses further supported the robustness of these findings. When lactate was replaced by BUN in the multivariable model (adjusted for vasoactive agent use), each 0.1 mmol/L decrease in iCa remained significantly associated with an increased risk of 28-day mortality (adjusted HR = 1.42, 95% CI, 1.17–1.73; *p* < 0.001). Moreover, in the expanded model that included iCa, vasoactive agent use, lactate, and BUN together, the association between iCa and mortality was essentially unchanged (adjusted HR = 1.50, 95% CI,.; *p* < 0.001), whereas BUN itself was not independently associated with mortality (*p* = 0.316). Collectively, these results indicate that the prognostic value of iCa is robust and does not depend on the specific choice of covariates used to adjust for disease severity (Supplementary Table S4).

### 3.4 Clinical characteristics of children with hypocalcemia at picu admission

**Figure 3:**
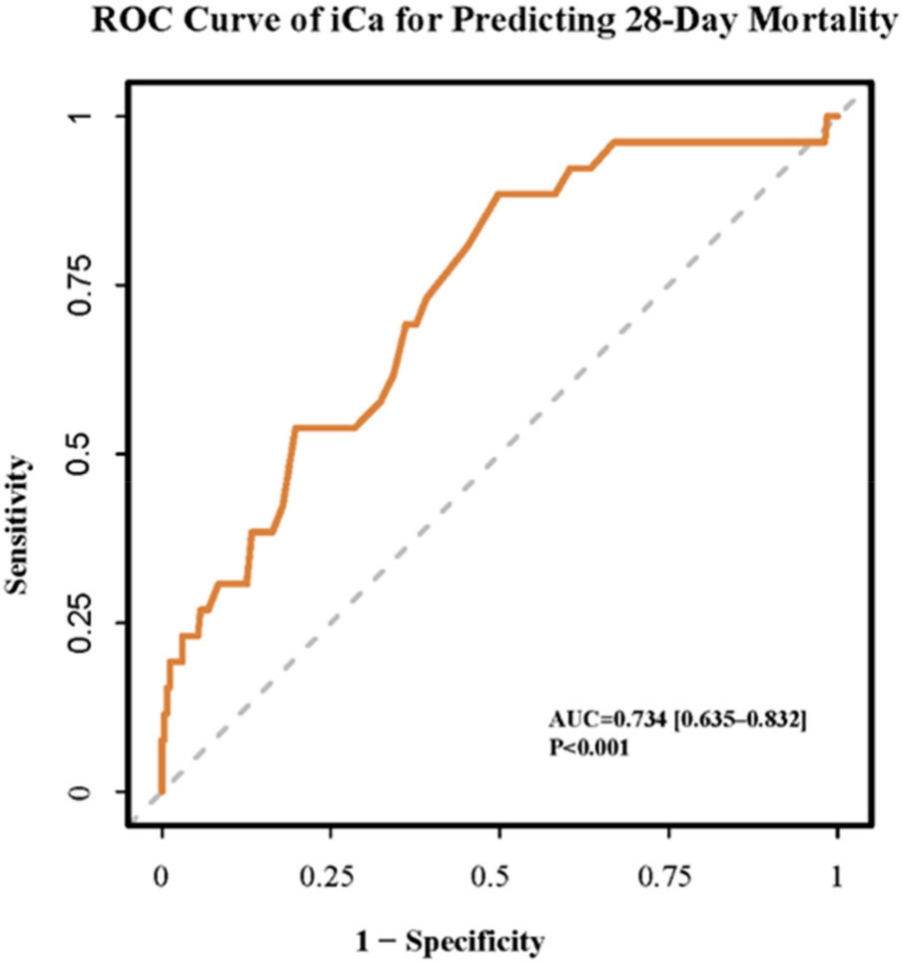
Performance of iCa in predicting 28-day mortality: A ROC curve analysis.

We next evaluated the discriminatory performance of serum iCa as a continuous biomarker for predicting 28-day mortality using ROC curve analysis. The area under the curve (AUC) was 0.734 (95% CI: 0.635–0.832, *P* < 0.001), indicating moderate discriminatory ability. Using the predefined clinical threshold for hypocalcemia (iCa < 1.15 mmol/L), the sensitivity and specificity for predicting 28-day mortality were 88.5% and 50.2%, respectively (Figure 3; Supplementary Table S5).

Patients with iCa < 1.15 mmol/L had significantly higher levels of lactate (2.55 vs. 1.70 mmol/L, *P* < 0.001), serum creatinine (55.00 vs. 43.00 μmol/L, *P* = 0.002), blood urea nitrogen (4.84 vs. 3.27 mmol/L, *P* < 0.001), aspartate aminotransferase (59.00 vs. 39.00 U/L, *P* = 0.005), C-reactive protein (50.65 vs. 21.00 mg/L, *P* = 0.002), and potassium (3.70 vs. 4.00 mmol/L, *P* = 0.001) than those with iCa ≥ 1.15 mmol/L. In addition, they had lower platelet counts (170.00 vs. 226.00 × 10⁹/L, *P* < 0.001) and lower albumin levels (29.09 vs. 32.79 g/L, *P* = 0.012). No significant differences were observed between the two groups in age, sex, SpO₂, white blood cell count, neutrophil count, alanine aminotransferase, total bilirubin, sodium, or chloride (Supplementary Table S6).

### 3.5 Incremental prognostic value of serum iCa

**Figure 4:**
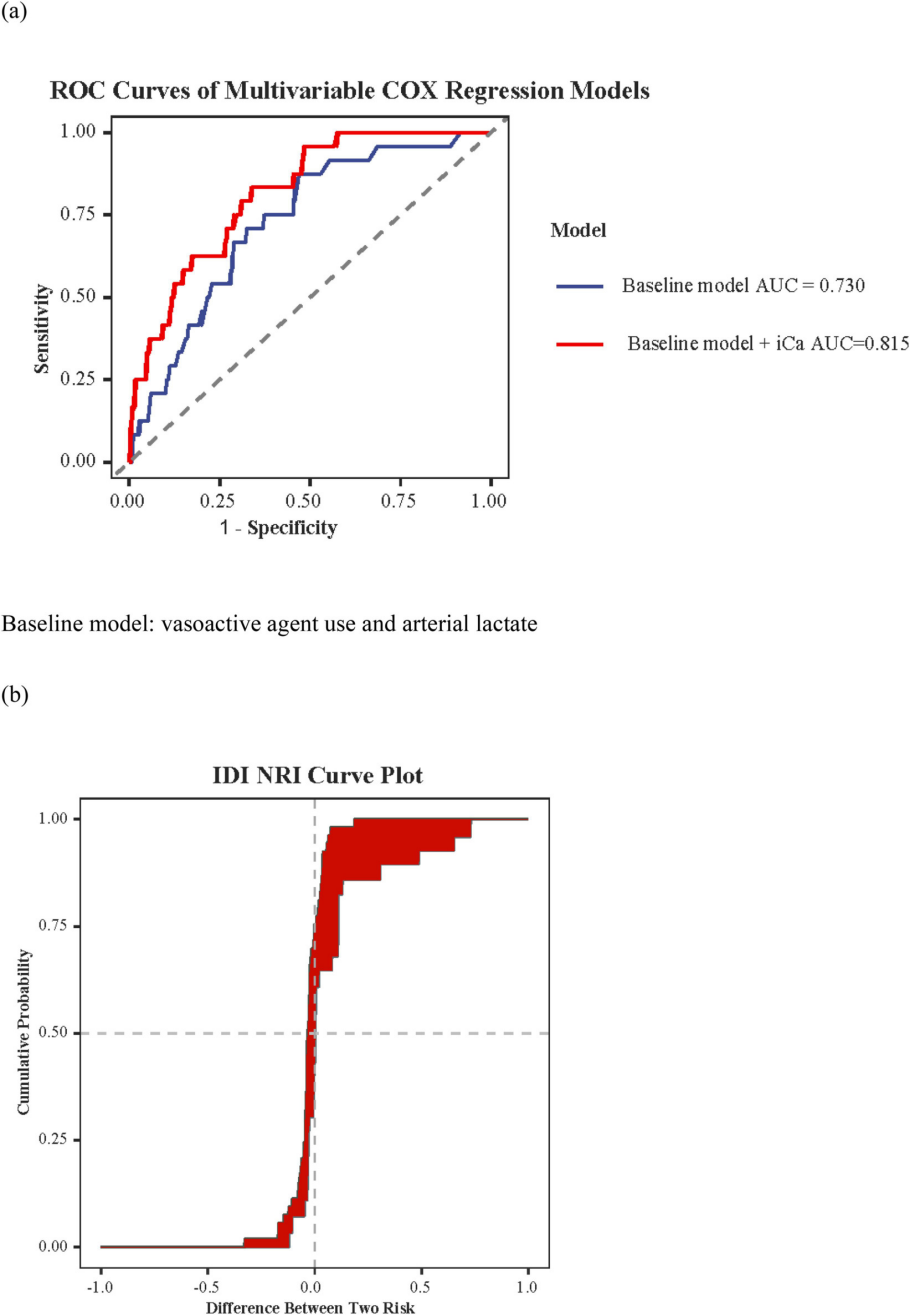
Discriminative performance and reclassification improvement of a multivariable COX model incorporating iCa. **(a)** ROC curves of multivariable Cox regression models: baseline vs. baseline plus iCa. Baseline model: vasoactive agent use and arterial lactate. **(b)** Distribution of risk differences between two models.

Given the distinct clinical features observed in the hypocalcemia group, we next assessed whether adding iCa to established clinical predictors could improve risk stratification. A baseline clinical prediction model incorporating vasoactive agent use and arterial lactate—two key indicators of disease severity—yielded an AUC of 0.730 (95% CI: 0.634–0.826) for predicting 28-day mortality. After inclusion of serum iCa, the AUC increased to 0.815 (95% CI: 0.739–0.892; Figure 4a). Although the improvement in AUC did not reach statistical significance according to the DeLong test (*p* = 0.126), reclassification analyses demonstrated a significant enhancement in risk discrimination (Figure 4b), with an integrated discrimination improvement (IDI) of 0.116 (*p* = 0.012), a net reclassification improvement (NRI) of 0.324 (*p* = 0.036), and a median improvement in predicted risk of 0.034 (*p* = 0.036; Supplementary Table S7).

### 3.6 Subgroup analyses and interaction effects

Subgroup analyses indicated that the association between lower serum iCa levels (per 0.1 mmol/L decrease) and increased mortality risk (HR = 1.51, 95% CI: 1.27–1.79) was consistent across age strata, with no significant interaction observed (p for interaction = 0.935). However, heterogeneity in the magnitude of this association was observed across subgroups stratified by disease severity. A significant association was identified among patients with initial lactate levels ≤ 4 mmol/L (HR = 1.69, 95% CI: 1.34–2.13, *p* < 0.001), whereas the association was attenuated and not statistically significant in those with lactate levels > 4 mmol/L (HR = 1.24, 95% CI: 0.92–1.67, *p* = 0.156), with a statistically significant interaction between groups (p for interaction = 0.014). A similar pattern was observed when stratifying by vasoactive agent use, although the interaction did not reach statistical significance (p for interaction = 0.061).

## 4 Discussion

In this single-center retrospective cohort study based on real-world PICU data, we systematically investigated the association between serum iCa levels at admission and 28-day all-cause mortality in children with sepsis. We found that hypocalcemia was highly prevalent in this population (50.8%), consistent with previous reports (18–21), and was significantly associated with an increased risk of short-term mortality. Notably, this association persisted after adjustment for key indicators of disease severity, including vasoactive agent use and lactate level, indicating that low iCa is not merely a nonspecific risk marker of critical illness but conveys independent prognostic information beyond conventional clinical indicators.

Our findings are highly consistent with previous studies conducted in critically ill children and neonates with sepsis. As early as 1987, Broner et al. reported that critically ill children with hypocalcemia at admission had a significantly higher mortality rate (12). Subsequently, in very low birth weight infants with sepsis, low iCa levels were independently associated with neonatal mortality and were identified as an independent predictor of adverse outcomes (14). The present study extends these findings to a broader pediatric sepsis population, confirming a strong association between admission iCa levels and short-term mortality. Compared with previous studies conducted in mixed critically ill populations where sepsis constituted only a subgroup (8, 11, 13, 16), our study focused on a well-defined cohort of children with sepsis through strict inclusion criteria based on the International Pediatric Sepsis Consensus Conference criteria and uniform analysis of admission iCa measurements, making the results more specific and clinically relevant. We observed that non-survivors exhibited more pronounced multi-organ dysfunction (higher creatinine, blood urea nitrogen, and transaminases) and inflammation (higher C-reactive protein), which aligns with the pathophysiology of septic shock (22–24); notably, low iCa levels were particularly prominent in these critically ill patients, further highlighting its potential value in assessing sepsis severity.

From a pathophysiological perspective, several mechanisms may explain the association between hypocalcemia and poor outcomes in sepsis. During sepsis, systemic inflammation and metabolic stress can induce massive catecholamine release, promoting the shift of extracellular calcium into the intracellular space. Simultaneously, inflammatory cytokines may suppress parathyroid hormone activity and impair vitamin D metabolism (10, 25–28). Calcium plays a critical role in myocardial contractility, vascular tone maintenance, and intracellular signal transduction (29). Theoretically, low iCa levels may directly lead to decreased myocardial contractility and vasodilation, thereby exacerbating hemodynamic derangements and tissue hypoperfusion. Our subgroup analysis showed that the prognostic value of iCa was more pronounced in patients with lower lactate levels (≤4 mmol/L) (30) or those not requiring vasoactive agents, which to some extent supports the aforementioned mechanism: the impact of hypocalcemia on hemodynamics may be more evident in patients with less severe disease.

Nevertheless, whether the association between hypocalcemia and poor outcomes represents a causal relationship or simply reflects disease severity remains uncertain. First, it is unclear whether admission iCa accurately reflects intracellular calcium dynamics, which are more directly linked to cellular function. Second, studies in critically ill adults have reported that exogenous calcium supplementation may not improve outcomes and may even be associated with adverse effects, which may be mediated by mechanisms such as mitochondrial dysfunction or pro-inflammatory effects (31, 32). This suggests that hypocalcemia may merely be a correlate of broader pathophysiological derangements—such as tissue hypoperfusion and systemic inflammation—rather than a direct causal factor. Therefore, given the observational design of this study, we are more inclined to regard iCa as a prognostic biomarker reflecting disease severity rather than an established causal therapeutic target.

Our ROC curve analysis demonstrated that, as a single biomarker, admission iCa exhibited moderate discriminatory ability for 28-day mortality. Notably, using the clinically recommended threshold for hypocalcemia (iCa < 1.15 mmol/L), iCa demonstrated high sensitivity (88.5%) for predicting 28-day mortality, supporting its feasibility for early risk stratification in pediatric sepsis.Based on this, we further evaluated its incremental value in clinical risk stratification. Reclassification analyses (integrated discrimination improvement [IDI] and net reclassification improvement [NRI]) showed that adding iCa to traditional clinical severity indicators (vasoactive agent use and lactate), more accurately reclassified patients' mortality risk. Although the improvement in AUC did not reach statistical significance—likely due to the limited sample size and number of outcome events—the significant improvements in IDI and NRI have important clinical implications. These findings suggest that iCa is not merely an epiphenomenon correlated with disease severity; rather, it provides incremental information that may assist clinicians in performing more refined risk stratification at the time of admission. Considering that iCa can be rapidly obtained upon admission (typically as part of blood gas analysis), its use as an early risk warning indicator offers high practicality and convenience, particularly in emergency departments and PICUs where rapid decision-making is essential.

However, our study also has several limitations. First, the retrospective single-center design and the use of ICD-10 codes to identify sepsis cases likely introduced selection bias, enriching our cohort with more severely ill children. In routine clinical practice, physicians are more likely to document “sepsis” in patients with overt organ dysfunction, whereas children with milder or early-stage sepsis often remain uncoded or are coded only by their primary etiology. This limits the generalizability of our findings to the full spectrum of pediatric sepsis. Moreover, we did not apply a prospective, literature-based case definition (e.g., IPSCC or Phoenix criteria) to confirm each diagnosis, which constitutes a major methodological limitation. Findings from this single-center study require external validation in multicenter prospective cohorts. Second, only the initial iCa measurement within 24 hours of admission was analyzed; we did not assess serial changes in iCa or the potential impact of calcium supplementation on outcomes. Third, although we excluded patients with known disorders of calcium metabolism, residual confounding from unmeasured factors (e.g., parathyroid hormone, vitamin D status) cannot be fully ruled out. Fourth, detailed data on comorbidities and infection sources were not systematically available, precluding analysis of their potential confounding or modifying effects. Fifth, comprehensive illness severity scores such as the pediatric Sequential Organ Failure Assessment (pSOFA) score were not included due to incomplete availability of key variables (e.g., PaO₂/FiO₂ ratio and neurological assessments), which may result in residual confounding despite adjustment for key indicators of organ dysfunction. In addition, data on respiratory support modalities (e.g., use of mechanical ventilation or level of respiratory assistance), which could serve as important proxies for respiratory dysfunction and overall illness severity, were not consistently available or retrievable from the current version of the database. This limitation further restricts our ability to fully account for the impact of respiratory failure on clinical outcomes. Finally, the relatively small sample size and limited number of death events reduced statistical power in subgroup analyses and constrained multivariable modeling to a parsimonious approach.

## 5 Conclusions

In summary, we observed that lower serum iCa levels at admission were independently associated with an increased risk of 28-day mortality in children with sepsis, after adjustment for key indicators of disease severity. As a readily available, inexpensive, and reproducible laboratory parameter, admission iCa may serve as a valuable adjunct to early risk stratification in pediatric sepsis. Future multicenter prospective studies are warranted to validate the prognostic value of admission iCa in pediatric sepsis and to explore its potential role in enhancing existing risk stratification tools.

## Data Availability

The datasets presented in this article are not readily available because the data analyzed in this study were derived from the publicly available Pediatric Intensive Care (PIC) database. Requests to access the datasets should be directed to http://pic.nbscn.org/.
