# Supplementary material for: Prognostic value of admission ionized calcium for short-term mortality in critically Ill children with sepsis: a single-center retrospective cohort study
Source: Front Pediatr. 2026 May 15;14:1793547. doi: 10.3389/fped.2026.1793547 (PMC13219297; doi:10.3389/fped.2026.1793547)
Supplement: Supplementary file 1 [file Table1.docx]

**Supplementary Table S1. Baseline Characteristics of Patients with Initial Lactate > 4 mmol/L Stratified by 28-Day Survival Status**

| **Characteristic** | **Total**  **(n=69)** | **Survivors**  **(n=58)** | | **Non-survivors**  **(n=11)** | **p-value** |
| --- | --- | --- | --- | --- | --- |
| **Demographics** |  |  | |  |  |
| Age (years) | 0.12 (0.01,0.34) | 0.10 (0.01,0.60) | | 0.13 (0.04,0.17) | 0.862 |
| Male, n (%) | 40 (59.97%) | 33 (56.90%) | | 7 (63.64%) | 0.750 |
| **Clinical Severity** |  |  | |  |  |
| Vasopressor Use, n (%) | 24 (38.10%) | 19 (35.85%) | | 5 (50.00%) | 0.398 |
| Lactate (mmol/L) | 6.00 (4.80,8.10) | 5.80 (4.70,8.05) | | 6.70 (5.30,9.95) | 0.241 |
| **Laboratory Parameters** |  |  | |  |  |
| WBC (10⁹/L) | 8.93 (4.32,13.69) | 9.07 (5.27,13.53) | | 3.76 (2.67,21.21) | 0.634 |
| Neutrophil (10⁹/L) | 5.39 (2.27,8.92) | 5.99 (2.74,9.01) | | 1.90 (0.97,5.09) | 0.042 |
| PLT (10⁹/L) | 144.00 (49.00,214.00) | 130.00 (44.75,207.25) | | 191.00 (83.50,270.5) | 0.228 |
| Creatinine (umol/L) | 69.00 (49.00,103.00) | 70.50 (50.25,98.75) | | 65.20 (44.50,107.00) | 0.793 |
| BUN (mmol/L) | 6.90 (4.25,9.35) | 6.45 (3.60,9.34) | | 8.05 (6.41,10.95) | 0.110 |
| Albumin (g/L) | 29.49 (7.57) | 29.87 (7.45) | | 27.48 (8.24) | 0.388 |
| ALT (U/L) | 29.00 (15.00,81.00) | 27.00 (15.00,72.25) | | 51.00 (21.50,136.5) | 0.207 |
| AST (U/L) | 108 (40.00,297.00) | 107  (40.00,238.25) | | 116.00 (70.00,646.50) | 0.222 |
| Total Bilirubin (umol/L) | 56.50 (19.3,138.7) | 55.75  (19.38,128.33) | | 93.3  (15.70,138.90) | 0.629 |
| CRP (mg/L), | 34.50 (12.52,77.44) | | 34.00 (11.52,70.89) | 43.50 (25.76,109.46) | 0.379 |
| K (mmol/L) | 4.30 (3.70,4.60) | | 4.30 (3.70,4.58) | 4.10 (3.75,5.05) | 0.640 |
| Na (mmol/L) | 136.00 (132.00,140.00) | | 135.00 (132.00,139.75) | 138.00 (125.5,139.5) | 0.366 |
| Cl (mmol/L) | 109.00 (105.00,112.00) | | 109.00 (104.00,112.00) | 108.00 (106.50,110.50) | 0.974 |
| iCa (mmol/L) | 1.08 (0.98,1.20) | | 1.09 (0.98,1.20) | 1.02(0.97,1.12) | 0.210 |

**Supplementary Table S2. Schoenfeld residual test for proportional hazards assumption in the multivariable Cox model.**

| **Variable** | **χ²** | **df** | **p-value** |
| --- | --- | --- | --- |
| iCa (per 0.1 mmol/L decrease) | 0.443 | 1 | 0.51 |
| Lactate (per 1 mmol/L increase) | 0.504 | 1 | 0.48 |
| Vasopressor use | 0.601 | 1 | 0.44 |
| GLOBAL | 1.107 | 3 | 0.78 |

**Supplementary Table S3. Variance inflation factor (VIF) for covariates in the multivariable Cox regression model.**

| **Covariate** | **VIF** |
| --- | --- |
| iCa (per 0.1 mmol/L decrease) | 1.099 |
| Lactate (per 1 mmol/L increase) | 1.231 |
| Vasopressor use | 1.133 |

**Supplementary Table S4. Sensitivity analyses for multivariable Cox regression models predicting 28-day mortality.**

| **Model** | **aHR for iCa**  **(per 0.1 mmol/L decrease)** | **95% CI** | **p-value** |
| --- | --- | --- | --- |
| Model 1 | 1.55 | 1.29–1.86 | <0.001 |
| Model 2 | 1.42 | 1.17–1.73 | <0.001 |
| Model 3 | 1.50 | 1.23–1.83 | <0.001 |

**Supplementary Table S5 Discriminative performance of admission serum ionized calcium (iCa) for 28-day mortality.**

| **Variable** | **AUC** | **95%CI** | **P value** | **Sensitivity** | **Specificity** |
| --- | --- | --- | --- | --- | --- |
| iCa | 0.734 | 0.635-0.832 | <0.001 | 0.885 | 0.502 |

**Sensitivity and specificity are reported at the predefined clinical threshold for hypocalcemia (iCa < 1.15 mmol/L). AUC was calculated for iCa as a continuous biomarker.”**

**Supplementary Table S6. Clinical Characteristics of Children With and Without Hypocalcemia at PICU Admission**

| **Characteristic** | **Hypocalcemia**  **(n=147)** | **Non-hypocalcemia (n=142)** | **p-value** |
| --- | --- | --- | --- |
| **Demographics** |  |  |  |
| Age (years) | 0.23 (0.01,2.26) | 0.19 (0.05,1.27) | 0.948 |
| Male, n (%) | 89 (60.54%) | 84 (59.15%) | 0.810 |
| **Clinical Severity** |  |  |  |
| Vasopressor Use, n (%) | 43 (29.86%) | 34 (25.56%) | 0.425 |
| Lactate (mmol/L) | 2.55 (1.55,4.75) | 1.70 (1.20,2.60) | <0.001 |
| **Laboratory Parameters** |  |  |  |
| WBC (10⁹/L) | 9.90 (3.80,15.85) | 9.94 (6.40,14.59) | 0.473 |
| Neutrophil (10⁹/L) | 6.13 (2.06,10.62) | 4.71 (2.54,9.73) | 0.604 |
| PLT (10⁹/L) | 170.00 (70.00,252.00) | 226.00 (117.00,348.00) | <0.001 |
| Creatinine (umol/L) | 55.00 (38.00,87.00) | 43.00 (36.00,60.00) | 0.002 |
| BUN (mmol/L) | 4.84 (3.04,7.95) | 3.27 (2.25,5.44) | <0.001 |
| Albumin (g/L) | 29.09 (6.42) | 32.79 (6.84) | 0.012 |
| ALT (U/L) | 23.00 (12.00,70.00) | 18.50 (10.00,43.00) | 0.095 |
| AST (U/L) | 59.00  (34.00,130.00) | 39.00.00 (24.00,82.00) | 0.005 |
| Total Bilirubin (umol/L) | 26.50  (8.70,97.00) | 13.95  (7.10,90.20) | 0.232 |
| CRP (mg/L), | 50.65 (13.71,109.50) | 21.00 (6.00,60.00) | 0.002 |
| K (mmol/L) | 3.70 (3.10,4.20) | 4.00 (3.50,4.30) | 0.001 |
| Na (mmol/L) | 135.00 (131.00,139.00) | 137.00 (134.00,140.00) | 0.050 |
| Cl (mmol/L) | 108.00 (104.00,112.00) | 109.00 (105.00,112.00) | 0.2333 |
| iCa (mmol/L) | 1.04 (0.95,1.09) | 1.24(1.19,1.29) | <0.001 |

**Supplementary Table S7 Incremental value indicators of serum ionized calcium in the prognostic model**

| Indicator | Estimate (95% CI) | P value |
| --- | --- | --- |
| IDI | 0.116 (0.025-0.261) | 0.012 |
| NRI | 0.324 (0.028-0.566) | 0.036 |
| Median_improvement | 0.034 (0.001-0.208) | 0.036 |
